# Performance of fine needle aspiration cytology and Ziehl-Neelsen staining technique in the diagnosis of tuberculosis lymphadenitis

**DOI:** 10.1186/s12879-024-09554-z

**Published:** 2024-06-25

**Authors:** Hawi Kumbi, Musa Mohammed Ali, Alegntaw Abate

**Affiliations:** 1 Department of Laboratory Science, Adama Hospital Medical College, Po Box 84, Adama, Ethiopia; 2 School of Medical Laboratory Science, College of Medicine and Health Sciences, Hawassa University, Po Box 1560, Hawassa, Ethiopia; 3 Department of Medical Laboratory Science, College of Health Sciences, Oda Bultum University, Po Box 226, Chiro, Ethiopia

**Keywords:** LTB, FNAC, ZN, Gene-Xpert, Sensitivity, Specificity, PPV, NPV

## Abstract

**Introduction:**

Proper diagnosis of tuberculosis (TB) lymphadenitis is critical for its treatment and prevention. Fine needle aspirate cytology (FNAC) is the mainstay method for the diagnosis of TB lymphadenitis in Ethiopia; however, the performance of FNAC has not been evaluated in the Eastern Region of Ethiopia. This study aimed to evaluate the performance of FNAC and Ziehl-Neelsen (ZN) staining compared with that of GeneXpert for the diagnosis of TB lymphadenitis.

**Methods:**

Fine needle aspiration (FNA) specimens collected from 291 patients suspected of having TB lymphadenitis were examined using FNAC, ZN, and GeneXpert to diagnose TB lymphadenitis. Gene-Xpert was considered the reference standard method for comparison. The sensitivity, specificity, positive predictive value (PPV), negative predictive value (NPV), and kappa coefficient were determined using SPSS version 25.

**Results:**

The sensitivity, specificity, PPV, and NPV of ZN for diagnosing TB lymphadenitis were 73.2%, 97.4%, 96.2%, and 80.1% respectively. There was poor agreement between ZN and GeneXpert (Kappa=-0.253). The sensitivity, specificity, PPV, and NPV of FNAC were 83.3%, 94.8%, 93.5%, and 86.3% respectively. There was moderate agreement between the FNAC and GeneXpert (Kappa = 0.785).

**Conclusion:**

The fine needle aspiration cytology (FNAC) is a more sensitive test for the diagnosis of TB lymphadenitis than ZN. The FNAC showed a moderate agreement with the GeneXpert assay. This study recommends the FNA GeneXpert MTB/RIF test in preference to FNAC for the diagnosis of TB lymphadenitis to avoid a missed diagnosis of smear-negative TB lymphadenitis.

## Introduction

Tuberculous lymphadenitis (TBL) is a chronic specific granulomatous inflammation that causes necrosis in lymph nodes [1]. It is often caused by reactivation of latent infection and is the most common manifestation of extrapulmonary tuberculosis (EPTB) [2, 3]. One-fourth of the world’s population is latently infected with *Mycobacterium tuberculosis* [4]. The World Health Organization (WHO) 2022 report shows that there were 10.6 million TB cases and 1.6 million deaths in 2021, compared to 10.1 million cases and 1.5 million deaths in 2020, with a 3.6% increase in the incidence rate [5].

Extrapulmonary TB accounts for 15–20% of all TB cases and accounts for 50% of HIV-confirmed cases [6]. TB lymphadenitis is observed in approximately 35% of EPTB patients [7]. Pulmonary TB and TB lymphadenitis are the most common forms of TB in the world [8]. In Ethiopia, approximately one-third of TB cases are attributed to TB lymphadenitis [9].

Tuberculous lymphadenitis is not part of the global TB response strategy because of its minor role in TB transmission [10]. However, there is evidence that TB lymphadenitis has a future impact on global TB control because of its ability to reactivate TB, its obscure location, its paucibacillary nature and its ability to be diagnosed at an advanced stage of the disease when complications are present [6, 11]. Therefore, proper diagnosis of TB lymphadenitis is critical for its treatment and prevention.

The WHO has endorsed the GeneXpert MTB/RIF assay as the fastest and most sensitive test compared to conventional methods, with greater feasibility for point-of-care implementation due to minimal infrastructure requirements [12]. Also in 2017, WHO confirmed the sensitivity similarity of the next-generation Xpert MTB/RIF ultra assay to solid culture with an improved limit of detection [13].

The microscopic diagnosis of pulmonary TB is essential in developing countries because it is inexpensive, rapid and sensitive, but the sensitivity is limited to 20–43% for EPTB patients [14]. FNAC is an important diagnostic method for EPTB [15]. In Ethiopia, FNAC is the mainstay method for the diagnosis of TB lymphadenitis; however, the performance of FNAC has not been evaluated in the Eastern Region of Ethiopia. This study aimed to evaluate the performance of FNAC and Ziehl-Neelsen (ZN) staining technique in combination with GeneXpert for the diagnosis of TB lymphadenitis at Adama Hospital Medical College (AHMC), Adama, Ethiopia.

## Materials and methods

### Study area and design

A hospital-based cross-sectional study was conducted among 291 patients suspected of having TB lymphadenitis at AHMC, Adama, Ethiopia, from May to August 2022. The city is located about 99 km due Southeast of Addis Ababa, the capital city of Ethiopia. It is located at latitude of 8° 54’ north and a longitude of 39° 27’ east. AHMC serves as a referral hospital for the people residing in the East Shewa Zone of the Oromia Regional State and adjacent regions. Sociodemographic and clinical data was collected using a structured preprepared format [16]. For the diagnosis of TBL, the ZN staining technique, FNAC, and GeneXpert were used. GeneXpert was considered the reference standard to which other methods were compared and the performance was determined accordingly.

### Sample collection and processing

Approximately 1.5-3 ml fine needle aspiration (FNA) samples were collected from the enlarged superficial lymph nodes using 22–23 gauge needles. One part of the sample was transferred to a sterile container with normal saline for GeneXpert and the remaining part was smeared on two different slides for cytological and Ziehl-Neelsen staining.

### Cytological diagnosis

The air-dried FNA smears were stained with Wright’s stain according to the SOPs [16] and examined microscopically by experienced pathologists. The FNAC shows up the epithelioid granulomas, and the epithelioid granulomas with multinucleated giant cells, caseous necrosis, degenerating inflammatory cells and liquefied necrotic materials were considered cytologically positive for tuberculosis [16, 17].

### Ziehl-Neelsen (ZN) staining

ZN staining was performed according to the SOP [16], and the stained smears were examined by microbiologists at AHMC under the oil-immersion objective (100*) of a light microscope. A minimum of 100 fields were scanned for negative results.

### GeneXpert MTB/RF assay

The GeneXpert MTB/RIF assay (Cepheid, CA, USA) was performed according to microbiology laboratory SOPs [16]: 2 ml of Gene-Xpert MTB/RIF Specimen Reagent Buffer was added to 1 ml of fine needle aspiration sample using a sterile pipette. The closed sample container was manually vortexed twice for 15 s, allowed to stand at room temperature for 10 min, vortexed after 10 min and allowed to stand for 5 min. Then 2 ml of the inactivated material was transferred to the test cartilage and the cartilage was loaded into the Gene-Xpert device. Finally, the results were interpreted by the Gene-Xpert diagnostic system from the measured fluorescence signals and automatically displayed after 2 h [16].

### Data analysis

The data were analyzed using SPSS version 25. The sensitivity, specificity, PPV, NPV, and Kappa coefficient were determined. The agreement between the tests and the reference method were evaluated using the Kappa value. The Kappa values 0-0.2, 0.21–0.39, 0.4–0.59, 0.6–0.79, 0.8–0.9, > 0.9 were interpreted as no agreement, minimal agreement, weak agreement, moderate agreement, strong agreement, and almost perfect agreement respectively [18].

### Ethical clearance

The study was approved by the Institutional Review Board of Hawassa University Medical College (reference number: IRB/148/14). The purpose and procedure of the study were explained to the study participants. Data were collected after written informed consent and/or assent was obtained from the study participants or the parents/guardians. Strict confidentiality was maintained throughout the study using only code.

## Results

A total of 291 FNAC samples were performed using cytology, ZN and GeneXpert methods. Among the 291 samples, about 138 (47.4%) were positive according to the reference standard method GeneXpert, 123(42.3%) were cytologically suggestive of TB and 105(36.1%) were positive for acid fast bacilli (AFB) in ZN staining microscopy. The sensitivity, specificity, PPV, and NPV of ZN for diagnosing TB lymphadenitis were 73.2%, 97.4%, 96.2%, and 80.1% respectively. There was poor agreement between ZN and GeneXpert (Kappa=-0.253). The sensitivity, specificity, PPV, and NPV of FNAC were 83.3%, 94.8%, 93.5%, and 86.3% respectively. There was moderate agreement between the FNAC and GeneXpert (Kappa = 0.785) (Table 1).

**Table 1:** Diagnostic performance of fine needle aspiration cytology and Ziehl-Neelsen staining technique compared to the GeneXpert reference standard in the diagnosis of tuberculosis lymphadenitis

Lab methods		Gene-Xpert		Total	Sensitivity (%) (95% CI)	Specificity (%) (95% CI)	PPV (%) (95% CI)	NPV (%) (95% CI)	Kappa test	*P*-value
		Pos	Neg							
ZN staining	Pos	101	4	105	73.2	97.4	96.2	80.1	-0.253	< 0.0001
	Neg	37	149	186						
Total		138	153	291						
FNAC	Pos	115	8	123	83.3	94.8	93.5	86.3	0.785	< 0.0001
	Neg	23	145	168						
Total		138	153	291						

## Discussion

One of the mainstays of TB care and control is accurate and timely diagnosis and effective treatment. In developed countries, a confirmed diagnosis of TB can only be made by culture or by finding a specific DNA sequence of the bacteria in a sputum sample for pulmonary TB and FNAC for extrapulmonary TB. However, in developing countries such as Ethiopia, these tests are not available in all areas of the country. In these countries, cost-effective techniques such as the ZN staining method and, in the case of EPTB, FNAC are very useful methods for detecting tuberculosis.

The sensitivity, specificity, PPV and NPV of ZN staining for the diagnosis of TB lymphadenitis in the present study were 73.2%, 97.4%, 96.2% and 80.1%, respectively. The sensitivity of ZN in the present study was lower than that reported from Tirupati, India (83.3%) [19] and India(91%) [20], while it was higher than that reported from Bangladesh (17.6%) [21] and Ethiopia (22.9%) [22]. However, the specificity of ZN staining in the present study (80.1%) was lower than that reported in Bangladesh (98.4%) [21], Ethiopia (92.4%) [22], Tirupati, India (88.9%) [19], India (90%) [20] and South Africa, 88.9% [23]. The percentage of agreement of ZN staining with GeneXpert was − 0.253 (Kappa test), indicating no agreement.

In the present study, the sensitivity, specificity, PPV and NPV of FNAC for the diagnosis of TB lymphadenitis were 83.3%, 94.8%, 93.5% and 86.3%, respectively. The sensitivity of FNAC in the present study was comparable to that reported Ethiopia (81%) [22] and Bangladesh (79.7%) [21] but lower than that reported in Egypt (90.9%) [24]. However, the specificity of FNAC in the present study (86.3%) was greater than that reported in Bangladesh (48.1%) [21], Ethiopia (50%) [22] and Egypt (67.2%) [24]. The percentage of agreement of FNAC with GeneXpert was 0.785 (Kappa test), indicating moderate agreement, which was similar to the results of a study conducted in India, where the percentage of agreement of FNAC with GeneXpert was 0.4 [25]. The highest sensitivity of TB lymphadenitis in ZN and FNAC in the present study may be due to the sample from only the TB lymphadenitis suspected patients.

## Conclusion

The fine needle aspiration cytology (FNAC) is a more sensitive test for the diagnosis of TB lymphadenitis than ZN. The FNAC showed a moderate agreement with the GeneXpert assay. This study recommends the FNA GeneXpert MTB/RIF test in preference to FNAC for the diagnosis of TB lymphadenitis to avoid a missed diagnosis of smear-negative TB lymphadenitis.

## Data Availability

The raw data sets used and/analyzed in the current study are available from the corresponding author upon reasonable request.

## Abbreviations

TB: Tuberculosis
TBL: TB lymphadenitis
EPTB: Extrapulmonary TB
FNAC: Fine Needle Aspirate Cytology
WHO: World Health Organization
SOP: Standard operating procedure
AHMC: Adama Hospital Medical College
PPV: Positive Predictive Value
NPV: Negative Predictive Value
